# Imaging of cervicothoracic junction anatomical variation in neurogenic thoracic outlet syndrome: A scoping review protocol

**DOI:** 10.1371/journal.pone.0352667

**Published:** 2026-07-02

**Authors:** Hugo O.H. Beaumont, George Slade, Hazel Brown, Tom J. Quick

**Affiliations:** 1 Southmead Hospital, North Bristol NHS Trust, Bristol, United Kingdom; 2 Musculoskeletal Research Unit, University of Bristol, Level 1 Learning and Research Building, Southmead Hospital, Bristol, United Kingdom; 3 Royal United Hospitals, Bath, United Kingdom; 4 South West Plexus Team, North Bristol NHS Trust, Bristol, United Kingdom; 5 University College London Centre for Nerve Engineering, London, United Kingdom; AIIMS: All India Institute of Medical Sciences, INDIA

## Abstract

**Objective:**

The objective of this scoping review is to systematically map the extent and nature of the literature on the use of imaging for assessing cervicothoracic junction anatomical variation in patients with neurogenic thoracic outlet syndrome.

**Introduction:**

Neurogenic thoracic outlet syndrome is a complex, disputed diagnosis that lacks a definitive gold-standard test, despite being the most common thoracic outlet syndrome subtype. Imaging is essential for identifying anatomical variations, such as cervical ribs, and excluding competing diagnoses. This review aims to clarify how imaging modalities are utilized to describe, classify, and assess these anatomical variations, and how these findings are explicitly linked to diagnosis or management.

**Selection criteria:**

This review will consider studies that include patients with neurogenic thoracic outlet syndrome and evaluate the role of imaging in assessing anatomical variations at the cervicothoracic junction. All study designs, including reviews, quantitative, and descriptive studies, will be considered. This review will exclude studies where the primary focus is the diagnosis of arterial or venous thoracic outlet syndrome using vascular imaging modalities.

**Methods:**

The search strategy will employ a three-step process, developed in consultation with a medical librarian, utilizing a final comprehensive search across multiple major databases (e.g., MEDLINE, CINAHL, Embase, Scopus) and gray literature sources. Studies published in the English language and from database inception will be included. Following screening by two independent reviewers, data will be extracted, synthesized descriptively, and presented in tabular and narrative format to map the identified evidence.

**Review Registration:**

Open Science Framework https://osf.io/hybg2

## Introduction

Thoracic outlet syndrome (TOS) is a debilitating and controversial group of disorders resulting from compression of one or more of the neurovascular structures that exit the thoracic outlet [1]. The most common type of which, neurogenic thoracic outlet syndrome (nTOS), involves compression of the brachial plexus and accounts for around 90–95 percent of cases [2]. Symptoms are varied, and can include weakness, numbness, paraesthesia, and pain, which can have a significant impact on quality of life [3]. Though surgeons have been operating on apparent TOS since 1861, [4] the investigation and diagnosis of the condition remains controversial [5]. Further, whilst nTOS is the most frequently seen subtype, its diagnosis may be the most challenging due to lack of readily observable vascular abnormalities [6].

As there is no single diagnostic test for nTOS, diagnosis relies on a combination of history, physical examination, electrodiagnostic (EDX) testing, and imaging [6]. Each of these, however, has limitations: symptoms vary between patients, [2] clinical tests lack specificity, [2] EDX findings are usually normal, [7] and imaging can appear unremarkable [8,9]. Some authors therefore distinguish between ‘true’ nTOS, characterized by objective findings such as a cervical rib and corresponding EDX abnormalities, and ‘disputed’ or ‘non-specific’ nTOS, which lacks these features but represents the vast majority of cases [10,11]. Anesthetic muscle blocks of the anterior scalene or pectoralis minor are also used as an adjunct to diagnosis, but lack specificity [12].

Given these diagnostic challenges, identifying anatomical predisposing factors provides important supplementary evidence to support the clinical diagnosis of nTOS, as well as offering a potential target of surgical intervention [13]. The pathophysiology is often described as an interaction between an underlying anatomical predisposition and a subsequent trigger, such as trauma or repetitive strain [9]. Relevant variations include osseous anomalies at the cervicothoracic junction (the transitional area between the cervical and thoracic spine), such as cervical ribs or elongated C7 transverse processes (TPs), and soft tissue variations like congenital fibrous bands or scalene muscle abnormalities [14]. A cervical rib (an additional rib arising from the seventh cervical vertebra) is the most frequently cited risk factor, with a prevalence of approximately 30% in TOS patients compared to about 1% in the general population [15]. However, most individuals with a cervical rib remain asymptomatic, highlighting the complexity of establishing causation [16]. An elongated C7 TP is less frequently implicated and is frequently omitted from reviews describing anatomical risk factors for TOS [17,18]. Although some case reports describe symptom resolution following resection, the causal relationship is less well-established than that of cervical ribs [19]. Many studies acknowledge this variation as a potential contributor, yet there remains a substantial evidence gap regarding its precise role in symptom development and best practice for imaging or classification. The presence of an elongated C7 TP may indeed represent an indicator of more widespread abnormality, rather than the actual direct cause of symptoms. The absence of consensus on which imaging findings are clinically relevant, together with the frequent appearance of anatomical variations in asymptomatic individuals, means that the role of imaging in the diagnosis and management of nTOS remains uncertain [20].

Given the complex anatomy, debated diagnostic criteria, and lack of consensus on optimal imaging for nTOS, a synthesis of existing evidence is needed. A scoping review is the most appropriate approach, as it systematically maps the breadth of literature on broad or complex topics, clarifies key concepts (such as classification systems for anatomical variation), and assesses the extent and nature of research activity. This review will therefore summarise current imaging approaches to the cervicothoracic junction in nTOS, examine how anatomical variations are described and classified across modalities, identify gaps in the evidence, and provide a foundation for developing standardized, evidence-based imaging guidelines.

A preliminary search of *MEDLINE*, the *Cochrane Database of Systematic Reviews* and *JBI Evidence Synthesis* was conducted and no current or underway systematic reviews or scoping reviews on the topic were identified. Although existing reviews have addressed the broader clinical diagnosis of thoracic outlet syndrome or focused on single risk factors such as cervical ribs, no scoping review to date has systematically mapped the full breadth of literature describing the imaging modalities used to identify and classify all cervicothoracic junction anatomical variations specifically in the context of nTOS.

### Review questions

1. How is the association between cervicothoracic junction anatomic variation and neurogenic thoracic outlet syndrome described in the literature?
2. What imaging modalities are used in the assessment of cervicothoracic junction anatomical variations in patients with neurogenic thoracic outlet syndrome?
3. How is imaging used to describe, classify, grade, and assess these anatomical variations in the literature?
4. How is imaging described as contributing to the diagnosis or management of neurogenic thoracic outlet syndrome?
5. Is there a recognized pattern or patterns of field defect in the cervicothoracic transition area that can be identified, and what is their link with C7 costal elements?

### Selection Criteria

#### Participants

This review will consider studies involving participants of any age who have been diagnosed with nTOS, inclusive of both ‘true’ and ‘disputed’ nTOS classifications. Studies that focus exclusively on vascular (arterial or venous) TOS without a neurogenic component will be excluded.

### Concept

The concept for this review is the use of any imaging modality (such as, but not limited to, radiography, computed tomography, magnetic resonance imaging, and ultrasonography) to identify, classify, assess, or report on anatomical variations of the cervicothoracic junction. This includes both osseous variations (e.g., cervical ribs, elongated C7 TPs) and soft tissue variations (e.g., congenital fibrous bands, scalene muscle anomalies, or aberrant vasculature causing neurogenic compression).

We will exclude studies that discuss nTOS diagnosis or management but do not report on specific imaging findings related to anatomical variations. Studies focusing exclusively on vascular imaging (e.g., angiography, arteriography) will also be excluded.

### Context

This review will consider evidence from any clinical or research setting where imaging for the investigation or diagnosis of nTOS is performed, including hospital departments, specialist clinics, and private imaging centers. There will be no restrictions based on geographic location or cultural context.

### Types of Sources

This scoping review will consider both experimental and quasi-experimental study designs including randomized controlled trials, non-randomized controlled trials, before and after studies and interrupted time-series studies. In addition, analytical observational studies including prospective and retrospective cohort studies, case-control studies and analytical cross-sectional studies will be considered for inclusion. This review will also consider descriptive observational study designs including case series, individual case reports and descriptive cross-sectional studies for inclusion.

Qualitative studies and text and opinion papers will also be considered for inclusion in this scoping review. In addition, systematic and scoping reviews that meet the inclusion criteria will also be considered.

## Methods

The proposed scoping review will be conducted in accordance with the JBI methodology for scoping reviews, [21] and reported in line with the Preferred Reporting Items for Systematic Reviews and Meta-Analyses extension for Scoping Reviews (PRISMA-ScR) [22]. This protocol has been registered in Open Science Framework (https://osf.io/hybg2).

### Search strategy

A three-step search strategy will be utilised in this review, developed in consultation with a medical librarian (JH) and refined using best practices. First, an initial limited search of MEDLINE (via Ovid) was undertaken to identify articles on the topic. The text words contained in the titles and abstracts of these articles, along with their associated index terms, were used to develop a comprehensive search strategy (see Appendix 1). The search strategy, including all identified keywords and index terms, will be adapted for each included database and information source. The reference lists of all included sources of evidence will be screened for additional studies (backward citation searching). Additionally, forward citation searching of highly relevant included studies will be performed using appropriate citation indexes.

Studies published in the English language will be included to maintain the accuracy of descriptive and conceptual terms. Studies published from database inception will be included to ensure comprehensive capture of the earliest literature relating to anatomical classifications.

The primary databases to be searched include MEDLINE (via Ovid), PubMed, Embase (Elsevier), Scopus, and CINAHL (EBSCOhost). While MEDLINE and PubMed share substantial overlap, both are included to ensure the exhaustive capture of all ‘ahead-of-print’ and non-indexed biomedical literature. The inevitable duplicate records resulting from this database overlap will be systematically identified and removed prior to screening.

Sources of grey literature will be searched to capture non-peer-reviewed and unpublished studies. For the purposes of this review, this includes academic theses, preprints, conference abstracts, and institutional reports. These sources will be identified using Google Scholar.

### Study/Source of evidence selection

Following the search, all identified citations will be collated and uploaded into Zotero 7.0.28 (Digital Scholar, Virginia, USA) and duplicates removed. Following a pilot test, titles and abstracts will then be screened by two independent reviewers (HOHB and GS) for assessment against the inclusion criteria for the review. Potentially relevant sources will be retrieved in full and their citation details imported into Rayyan [23]. The full text of selected citations will be assessed in detail against the inclusion criteria by two independent reviewers. Consistent with the JBI methodology for scoping reviews, critical appraisal of individual sources of evidence will not be performed [21]. Reasons for exclusion of sources of evidence at full text that do not meet the inclusion criteria will be recorded and reported in the scoping review. Any disagreements that arise between the reviewers at each stage of the selection process will be resolved through discussion, or with an additional reviewer. The results of the search and the study inclusion process will be reported in full in the final scoping review and presented in a PRISMA flow diagram [24].

### Data extraction

Data will be extracted from papers included in the scoping review by two independent reviewers (HOHB and GS) using a data extraction tool developed by the reviewers. The data extracted will include specific details about the participants, concept, context, study methods and key findings relevant to the review questions.

A draft extraction form is provided (see Appendix 2). The draft data extraction tool will be modified and revised as necessary during the process of extracting data from each included evidence source. Modifications will be detailed in the scoping review. Any disagreements that arise between the reviewers will be resolved through discussion, or with an additional reviewer. If appropriate, authors of papers will be contacted to request missing or additional data, where required.

### Data analysis and presentation

Data analysis and presentation will involve a descriptive numerical analysis and a thematic summary, presented in graphical, diagrammatic, and tabular formats to align with the review’s objectives. Descriptive charts will illustrate the distribution of studies by publication year, country of origin, and study design. A main table will detail the characteristics of all included sources. A cross-tabulation matrix will be used to map the anatomical variations against the imaging modalities employed. Further descriptive tables will summarize identified classification systems and the reported role of imaging. A narrative summary will accompany all results, synthesizing the charted and tabulated data to describe how the findings relate to the review’s questions and objectives. This presentation plan may be refined as data are extracted, and the final review will adhere to the PRISMA-ScR guidance [22].

### Study status and timeline

At the time of submission (May 2026), preliminary searches have been conducted to inform the search strategy, but no formal data screening or extraction has commenced. The study is scheduled to proceed according to the following timeline:

- **Record screening:** We anticipate the completion of title and abstract screening, as well as full-text review, by June 2026.
- **Data extraction:** The data charting process is expected to be completed by July 2026.
- **Results:** The final synthesis of results and drafting of the review manuscript is expected to be completed by September 2026.

## Supporting information

S1 File Appendix 1: Search Strategy. (PDF)

S2 File Appendix 2: Data Extraction Tool. (PDF)

S3 File PRISMA-P 2015 Checklist. (DOCX)
