# Supplementary material for: Imaging of cervicothoracic junction anatomical variation in neurogenic thoracic outlet syndrome: A scoping review protocol
Source: PLoS One. 2026 Jul 2;21(7):e0352667. doi: 10.1371/journal.pone.0352667 (PMC13327147; doi:10.1371/journal.pone.0352667)
Supplement: S1 File — (PDF) [file pone.0352667.s001.pdf]

Database: Ovid MEDLINE(R) ALL <1946 to October 22, 2025>

Search Strategy:

1 Thoracic Outlet Syndrome/ and neuro*.ti,ab,kf. (844)

2 (neuro* adj3 thoracic outlet syndrome).ti,ab,kf. (438)

3 ((neuro* adj3 TOS) or (nTOS or n-TOS)).ti,ab,kf. (486)

4 1 or 2 or 3 (1129)

5 Cervicothoracic junction.ti,ab,kf. (597)

6 (C7-T1 adj4 (spine or spinal)).ti,ab,kf. (71)

7 (C7 adj4 (spinous or transverse)).ti,ab,kf. (278)

8 "Elongated C7".ti,ab,kf. (15)

9 (anatom* adj4 vari*).ti,ab,kf. (30472)

10 (transitional adj4 (anatomy or vertebra*)).ti,ab,kf. (577)

11 (cervical adj2 rib*).ti,ab,kf. (964)

12 scalene muscle*.ti,ab,kf. (459)

13 (fibrous adj4 band*).ti,ab,kf. (1323)

14 (congenital adj4 (abnormal* or anomal* or malform*)).ti,ab,kf. (72354)

15 (anatom* adj4 (abnormal* or anomal* or malform*)).ti,ab,kf. (11113)

16 (bone adj4 (abnormal* or anomal* or malform*)).ti,ab,kf. (10388)

17 ((bone or bony) adj4 ossifi*).ti,ab,kf. (1890)

18 (brachial plexus adj4 (compress* or abnormal* or anomal* or malform* or entrap*)).ti,ab,kf. (486)

19 ((neuro* or neural or nerve*) adj4 (compress* or abnormal* or anomal* or malform* or entrap*)).ti,ab,kf. (65114)

20 ((muscle* or muscular) adj4 (compress* or abnormal* or anomal* or malform*)).ti,ab,kf. (10782)

21 5 or 6 or 7 or 8 or 9 or 10 or 11 or 12 or 13 or 14 or 15 or 16 or 17 or 18 or 19 or 20 (197336)

22 4 and 21 (606)

23 exp diagnostic imaging/ (3083590)

24 "Imaging".ti,ab,kf. (1253923)

25 diagnos*.ti,ab,kf. (3547357)

26 ("Magnetic resonance imaging" or MRI).ti,ab,kf. (560254)

27 ("Magnetic resonance neurography" or "MR neurography").ti,ab,kf. (759)

28 (Comput* adj3 tomography).ti,ab,kf. (419790)

29 X-ray.ti,ab,kf. (457814)

30 Radiography.ti,ab,kf. (79742)

31 Sonography.ti,ab,kf. (37016)

32 Ultrasound.ti,ab,kf. (368550)

33 23 or 24 or 25 or 26 or 27 or 28 or 29 or 30 or 31 or 32 (6889433)

34 22 and 33 (396)
