# Supplementary material for: Imaging of cervicothoracic junction anatomical variation in neurogenic thoracic outlet syndrome: A scoping review protocol
Source: PLoS One. 2026 Jul 2;21(7):e0352667. doi: 10.1371/journal.pone.0352667 (PMC13327147; doi:10.1371/journal.pone.0352667)
Supplement: S2 File — (PDF) [file pone.0352667.s002.pdf]

General Source Information	Extraction Details	Data
Author(s)		
Year		
Country of Origin	(Country where the research was conducted/published from)	
Study Design	(e.g., Sys review, Scoping review, Cohort, Case Series, Cross-sectional, etc.)	
Participant Details	(Population description, sample size, age range, sex)	
Aims/Purpose of Source	(Briefly state the main objective of the paper)	
**Research Questions**		
**1. How is the association between anatomical variation and nTOS described?**		
Description of association	(Summarise or quote how the source describes the link between the variant and the syndrome)	
Stated pathophysiological mechanism:	(e.g., direct nerve compression, fibrous bands, repetitive friction, vascular compromise leading to neural effects)	
**2. What imaging modalities are used?**		
Imaging modalities:	(e.g., XR, CT, MRI, USS, etc)	
Justification for modality choice:	(Note if the authors state why a particular modality was used)	
**3. How is imaging used to describe, classify, grade, and assess variations?**		
Specific anatomical variants identified:	(List all variants discussed, e.g., cervical rib, elongated C7 transverse process, anomalous scalene muscle)	
Classification system(s) used/mentioned:	(Note any specific grading or classification systems)	
Key imaging signs, descriptors, or measurements:	(e.g., costoclavicular angle, presence of fibrous bands, specific measurements of a transverse process)	
**4. How does imaging contribute to diagnosis or management?**		
Stated role in diagnosis:	(e.g., Initial investigation, confirmation of clinical suspicion, exclusion of other pathologies)	
Stated role in management	(e.g., Pre-op planning, patient selection for surgery, monitoring response to conservative treatment)	
**5. Is there a recognised pattern or patterns of field defect and what is their link with C7 costal elements?**		
Which co-occuring anomalies are reported?	(e.g., muscle, fibrous, TP, SP, other bony structures)	
How is this relationship described or explained?	(e.g. causative, etc)	
**General Notes**	(Capture any other relevant information, key author conclusions, or limitations noted in the source that are pertinent to the scoping review's objectives)	
