# Supplementary material for: Imaging of cervicothoracic junction anatomical variation in neurogenic thoracic outlet syndrome: A scoping review protocol
Source: PLoS One. 2026 Jul 2;21(7):e0352667. doi: 10.1371/journal.pone.0352667 (PMC13327147; doi:10.1371/journal.pone.0352667)
Supplement: Attachment — Submitted filename: Response To Reviewers.docx [file pone.0352667.s004.docx]

**Response To Reviewers**

The authors would like to sincerely thank the reviewers for their time and careful consideration of our manuscript. We completely agree that factors such as arm positioning, dynamic imaging protocols, and interobserver reliability are essential in the evaluation of neurogenic thoracic outlet syndrome. Because this is a scoping review designed to map the breadth of the literature rather than evaluate diagnostic accuracy, our primary review questions were kept intentionally broad to capture all of these elements.

With regards to some specific points of revision:

- The term “inclusion criteria” has been changed to “selection criteria” as suggested on lines 48 and 140 to describe the section more accurately.
- The reference to “unpublished studies” in the “Search strategy” section has been adjusted to further clarify as advised.
- Search methodology has been revised include PubMed alongside MEDLINE, which will guarantee the capture of recent, unindexed, and 'ahead-of-print' biomedical literature. We have also clarified in the manuscript that any inevitable duplicate records resulting from the overlap between these databases will be systematically managed and removed. Furthermore, the entire section has been thoroughly reviewed and rewritten to improve grammatical clarity.

We understand reviewer #2’s concerns regarding the absence of final data, included paper counts, and study results. However, I believe there may have been a misunderstanding due to the ambiguity of the term “protocol”, which in this case does not describe an imaging protocol, or any suggestion of which imaging modalities to use in thoracic outlet syndrome. We wish to respectfully clarify that this manuscript was submitted specifically as a **Study Protocol**, rather than a completed research article. In accordance with PLOS ONE’s editorial policies for Protocols, this article type is designed to detail the methodological roadmap for a future study and is not expected to include final data extraction or results.

We hope this clarifies the scope and intent of our submission, and that both reviewers are happy with our revised submission.

Kind regards,

Mr Hugo OH Beaumont
