# Supplementary material for: Imaging of cervicothoracic junction anatomical variation in neurogenic thoracic outlet syndrome: A scoping review protocol
Source: PLoS One. 2026 Jul 2;21(7):e0352667. doi: 10.1371/journal.pone.0352667 (PMC13327147; doi:10.1371/journal.pone.0352667)
Supplement: Peer review history

# Peer review: Decision Letter 0

**DOI:** 10.1371/journal.pone.0352667.r001

**Authors:** Priti Chaudhary

15 Apr 2026

PONE-D-26-05244

Imaging of cervicothoracic junction anatomical variation in neurogenic thoracic outlet syndrome: a scoping review protocol

PLOS One

Dear Dr. Beaumont,

Thank you for submitting your manuscript to PLOS ONE. After careful consideration, we feel that it has merit but does not fully meet PLOS ONE’s publication criteria as it currently stands. Therefore, we invite you to submit a revised version of the manuscript that addresses the points raised during the review process.

==============================

**ACADEMIC EDITOR:**

The authors must address all queries raised by the reviewers.

Please ensure that your decision is justified on PLOS ONE’s publication criteria and not, for example, on novelty or perceived impact.

For Lab, Study and Registered Report Protocols: These article types are not expected to include results but may include pilot data.

==============================

Please submit your revised manuscript by May 30 2026 11:59PM. If you will need more time than this to complete your revisions, please reply to this message or contact the journal office at plosone@plos.org. When you're ready to submit your revision, log on to https://www.editorialmanager.com/pone/ and select the 'Submissions Needing Revision' folder to locate your manuscript file.

Please include the following items when submitting your revised manuscript:

- A letter that responds to each point raised by the academic editor and reviewer(s). You should upload this letter as a separate file labeled 'Response to Reviewers'.
- A marked-up copy of your manuscript that highlights changes made to the original version. You should upload this as a separate file labeled 'Revised Manuscript with Track Changes'.
- An unmarked version of your revised paper without tracked changes. You should upload this as a separate file labeled 'Manuscript'.

If you would like to make changes to your financial disclosure, please include your updated statement in your cover letter. Guidelines for resubmitting your figure files are available below the reviewer comments at the end of this letter.

If applicable, we recommend that you deposit your laboratory protocols in protocols.io to enhance the reproducibility of your results. Protocols.io assigns your protocol its own identifier (DOI) so that it can be cited independently in the future. For instructions see: https://journals.plos.org/plosone/s/submission-guidelines#loc-laboratory-protocols. Additionally, PLOS ONE offers an option for publishing peer-reviewed Lab Protocol articles, which describe protocols hosted on protocols.io. Read more information on sharing protocols at https://plos.org/protocols?utm_medium=editorial-email&utm_source=authorletters&utm_campaign=protocols.

As the corresponding author, your ORCID iD is verified in the submission system and will appear in the published article. PLOS supports the use of ORCID, and we encourage all coauthors to register for an ORCID iD and use it as well. Please encourage your coauthors to verify their ORCID iD within the submission system before final acceptance, as unverified ORCID iDs will not appear in the published article. *Only*  the individual author can complete the verification step; PLOS staff *cannot*  verify ORCID iDs on behalf of authors.

We look forward to receiving your revised manuscript.

Kind regards,

Priti Chaudhary, M.S.

Academic Editor

PLOS One

Journal Requirements:

When submitting your revision, we need you to address these additional requirements.

1. Please ensure that your manuscript meets PLOS ONE's style requirements, including those for file naming. The PLOS ONE style templates can be found at

https://journals.plos.org/plosone/s/file?id=wjVg/PLOSOne_formatting_sample_main_body.pdf and

https://journals.plos.org/plosone/s/file?id=ba62/PLOSOne_formatting_sample_title_authors_affiliations.pdf

2. Thank you for stating the following in the Acknowledgments Section of your manuscript:

“No funding was gained for this review.”

We note that you have provided funding information that is not currently declared in your Funding Statement. However, funding information should not appear in the Acknowledgments section or other areas of your manuscript. We will only publish funding information present in the Funding Statement section of the online submission form.

Please remove any funding-related text from the manuscript and let us know how you would like to update your Funding Statement. Currently, your Funding Statement reads as follows:

“The author(s) received no specific funding for this work.”

Please include your amended statements within your cover letter; we will change the online submission form on your behalf.

3. If the reviewer comments include a recommendation to cite specific previously published works, please review and evaluate these publications to determine whether they are relevant and should be cited. There is no requirement to cite these works unless the editor has indicated otherwise.

[Note: HTML markup is below. Please do not edit.]

Reviewers' comments:

Reviewer's Responses to Questions

**Comments to the Author**

1. Does the manuscript provide a valid rationale for the proposed study, with clearly identified and justified research questions?

The research question outlined is expected to address a valid academic problem or topic and contribute to the base of knowledge in the field.

Reviewer #1: Yes

Reviewer #2: Partly

**********

2. Is the protocol technically sound and planned in a manner that will lead to a meaningful outcome and allow testing the stated hypotheses?

The manuscript should describe the methods in sufficient detail to prevent undisclosed flexibility in the experimental procedure or analysis pipeline, including sufficient outcome-neutral conditions (e.g. necessary controls, absence of floor or ceiling effects) to test the proposed hypotheses and a statistical power analysis where applicable. As there may be aspects of the methodology and analysis which can only be refined once the work is undertaken, authors should outline potential assumptions and explicitly describe what aspects of the proposed analyses, if any, are exploratory.

Reviewer #1: Yes

Reviewer #2: Partly

**********

3. Is the methodology feasible and described in sufficient detail to allow the work to be replicable?

Descriptions of methods and materials in the protocol should be reported in sufficient detail for another researcher to reproduce all experiments and analyses. The protocol should describe the appropriate controls, sample size calculations, and replication needed to ensure that the data are robust and reproducible.

Reviewer #1: Yes

Reviewer #2: Yes

**********

4. Have the authors described where all data underlying the findings will be made available when the study is complete?

The PLOS Data policy requires authors to make all data underlying the findings described in their manuscript fully available without restriction, with rare exception, at the time of publication. The data should be provided as part of the manuscript or its supporting information, or deposited to a public repository. For example, in addition to summary statistics, the data points behind means, medians and variance measures should be available. If there are restrictions on publicly sharing data—e.g. participant privacy or use of data from a third party—those must be specified.

Reviewer #1: Yes

Reviewer #2: Yes

**********

5. Is the manuscript presented in an intelligible fashion and written in standard English?

PLOS ONE does not copyedit accepted manuscripts, so the language in submitted articles must be clear, correct, and unambiguous. Any typographical or grammatical errors should be corrected at revision, so please note any specific errors here.

Reviewer #1: Yes

Reviewer #2: Yes

**********

6. Review Comments to the Author

Please use the space provided to explain your answers to the questions above and, if applicable, provide comments about issues authors must address before this protocol can be accepted for publication. You may also include additional comments for the author, including concerns about research or publication ethics.

You may also provide optional suggestions and comments to authors that they might find helpful in planning their study.

(Please upload your review as an attachment if it exceeds 20,000 characters)

Reviewer #1: Find below some comments that may improve the quality of the manuscripts

-The title could be made clearer to better reflect the intent of the review. Consider incorporating the phrase “Imaging Modalities/Approaches” to improve specificity.

-Lines 48 and 140 the term “Inclusion Criteria” should be revised to “Selection Criteria”, as both inclusion and exclusion criteria are described in this section.

-Line 180 Please clarify what is meant by “unpublished studies.” Does this include non–peer-reviewed studies? How does this differ from gray literature sources?

-Lines 181–190 The rationale for excluding PubMed is unclear. Please review the grammar and improve clarity. Additionally, consider expanding this section to clearly justify the choice of databases and search strategy.

Other thing you need to pay attention to

-What are the key anatomical structures at the cervicothoracic junction relevant to nTOS?

-Which imaging modalities are most appropriate for evaluating the cervicothoracic junction in nTOS?

-What are the strengths and limitations of each modality in detecting.

-Factor affecting imaging e.g. how does arm positioning (abduction, elevation) affect imaging findings?

-What is the role of dynamic imaging in diagnosing nTOS and are there standardized protocols for positional imaging?

-How are anatomical variations quantified or classified and How reproducible are imaging findings (interobserver reliability)?

Best regards

Reviewer #2: It is really hard work with so many data collections and analysis though I could not figure out how many papers you have studied and how many you have included.

To my concern I did not find a recommended protocol I concluded that you just listed the stepwise process you have followed to obtain your results.

I realized that it is the first one to be conducted in this area of research but the question here do you recommend you fellows to follow the same process you have followed if they conducted similar researches??!! Is your protocol a valid protocol??!!

**********

7. PLOS authors have the option to publish the peer review history of their article (what does this mean?). If published, this will include your full peer review and any attached files.

If you choose “no”, your identity will remain anonymous but your review may still be made public.

**Do you want your identity to be public for this peer review?**  For information about this choice, including consent withdrawal, please see our Privacy Policy.

Reviewer #1: **Yes:**  Dr Lawan Hassan Adamu

Reviewer #2: No

**********

[NOTE: If reviewer comments were submitted as an attachment file, they will be attached to this email and accessible via the submission site. Please log into your account, locate the manuscript record, and check for the action link "View Attachments". If this link does not appear, there are no attachment files.]

To ensure your figures meet our technical requirements, please review our figure guidelines: https://journals.plos.org/plosone/s/figures

You may also use PLOS’s free figure tool, NAAS, to help you prepare publication quality figures: https://journals.plos.org/plosone/s/figures#loc-tools-for-figure-preparation.

NAAS will assess whether your figures meet our technical requirements by comparing each figure against our figure specifications.

# Author comment: Author response to Decision Letter 1

**DOI:** 10.1371/journal.pone.0352667.r002

29 May 2026

Response To Reviewers

The authors would like to sincerely thank the reviewers for their time and careful consideration of our manuscript. We completely agree that factors such as arm positioning, dynamic imaging protocols, and interobserver reliability are essential in the evaluation of neurogenic thoracic outlet syndrome. Because this is a scoping review designed to map the breadth of the literature rather than evaluate diagnostic accuracy, our primary review questions were kept intentionally broad to capture all of these elements.

With regards to some specific points of revision:

• The term “inclusion criteria” has been changed to “selection criteria” as suggested on lines 48 and 140 to describe the section more accurately.

• The reference to “unpublished studies” in the “Search strategy” section has been adjusted to further clarify as advised.

• Search methodology has been revised include PubMed alongside MEDLINE, which will guarantee the capture of recent, unindexed, and 'ahead-of-print' biomedical literature. We have also clarified in the manuscript that any inevitable duplicate records resulting from the overlap between these databases will be systematically managed and removed. Furthermore, the entire section has been thoroughly reviewed and rewritten to improve grammatical clarity.

We understand reviewer #2’s concerns regarding the absence of final data, included paper counts, and study results. However, I believe there may have been a misunderstanding due to the ambiguity of the term “protocol”, which in this case does not describe an imaging protocol, or any suggestion of which imaging modalities to use in thoracic outlet syndrome. We wish to respectfully clarify that this manuscript was submitted specifically as a Study Protocol, rather than a completed research article. In accordance with PLOS ONE’s editorial policies for Protocols, this article type is designed to detail the methodological roadmap for a future study and is not expected to include final data extraction or results.

We hope this clarifies the scope and intent of our submission, and that both reviewers will be satisfied with our revised submission.

Kind regards,

Mr Hugo OH Beaumont

Attachment Submitted filename: Response To Reviewers.docx

# Editor report: Decision Letter 1

**DOI:** 10.1371/journal.pone.0352667.r003

**Authors:** Priti Chaudhary

15 Jun 2026

Imaging of cervicothoracic junction anatomical variation in neurogenic thoracic outlet syndrome: a scoping review protocol

PONE-D-26-05244R1

Dear Dr. Hugo Oscar Hamilton Beaumont,

We’re pleased to inform you that your manuscript has been judged scientifically suitable for publication and will be formally accepted for publication once it meets all outstanding technical requirements.

Within one week, you’ll receive an e-mail detailing the required amendments. When these have been addressed, you’ll receive a formal acceptance letter and your manuscript will be scheduled for publication.

An invoice will be generated when your article is formally accepted. Please note, if your institution has a publishing partnership with PLOS and your article meets the relevant criteria, all or part of your publication costs will be covered. Please make sure your user information is up-to-date by logging into Editorial Manager at Editorial Manager® and clicking the ‘Update My Information' link at the top of the page. For questions related to billing, please contact billing support.

If your institution or institutions have a press office, please notify them about your upcoming paper to help maximize its impact. If they’ll be preparing press materials, please inform our press team as soon as possible -- no later than 48 hours after receiving the formal acceptance. Your manuscript will remain under strict press embargo until 2 pm Eastern Time on the date of publication. For more information, please contact onepress@plos.org.

Kind regards,

Priti Chaudhary, M.S.

Academic Editor

PLOS One

Additional Editor Comments (optional):

Reviewers' comments:

# Editor report: Acceptance letter

**DOI:** 10.1371/journal.pone.0352667.r004

**Authors:** Priti Chaudhary

PONE-D-26-05244R1

PLOS One

Dear Dr. Beaumont,

I'm pleased to inform you that your manuscript has been deemed suitable for publication in PLOS One. Congratulations! Your manuscript is now being handed over to our production team.

At this stage, our production department will prepare your paper for publication. This includes ensuring the following:

* All references, tables, and figures are properly cited

* All relevant supporting information is included in the manuscript submission,

* There are no issues that prevent the paper from being properly typeset

You will receive further instructions from the production team, including instructions on how to review your proof when it is ready. Please keep in mind that we are working through a large volume of accepted articles, so please give us a few days to review your paper and let you know the next and final steps.

Lastly, if your institution or institutions have a press office, please let them know about your upcoming paper now to help maximize its impact. If they'll be preparing press materials, please inform our press team within the next 48 hours. Your manuscript will remain under strict press embargo until 2 pm Eastern Time on the date of publication. For more information, please contact onepress@plos.org.

You will receive an invoice from PLOS for your publication fee after your manuscript has reached the completed accept phase. If you receive an email requesting payment before acceptance or for any other service, this may be a phishing scheme. Learn how to identify phishing emails and protect your accounts at https://explore.plos.org/phishing.

If we can help with anything else, please email us at customercare@plos.org.

Thank you for submitting your work to PLOS ONE and supporting open access.

Kind regards,

PLOS ONE Editorial Office Staff

on behalf of

Dr. Priti Chaudhary

Academic Editor

PLOS One
